# Component of oligomeric Golgi complex 1 deficiency leads to hypoglycemia: a case report and literature review

**DOI:** 10.1186/s12887-021-02922-7

**Published:** 2021-10-08

**Authors:** Yizhou Huang, Han Dai, Gangyi Yang, Lili Zhang, Shiyao Xue, Min Zhu

**Affiliations:** 1 Department of Endocrinology, The Second Affiliated Hospital, Chongqing Medical University, Yuzhong Dist, Chongqing, 404100 People’s Republic of China; 2 Department of Endocrinology, Children’s Hospital of Chongqing Medical University, Chongqing, China

**Keywords:** CDG-IIg, *COG1* gene, case report, mutation, glycosylation

## Abstract

**Background:**

Congenital disorders of glycosylation (CDG) are a group of metabolic diseases with clinical and genetic heterogeneity, and CDG-IIg is one of the rare reported types of CDG. The aim of this study is to report the clinical manifestations and gene-phenotype characteristics of a rare case of CDG caused by a *COG1* gene mutation and review literatures of CDG disease.

**Case presentation:**

The patient was male, and the main clinical symptoms were developmental retardation, convulsion, strabismus, and hypoglycemia, which is rarely reported in CDG-IIg. We treated the patient with glucose infusion and he was recovered from hypoglycemia. Genetic analysis showed that the patient carried the heterozygous intron mutation c.1070 + 3A > G (splicing) in the coding region of the *COG1* gene that was inherited from the mother, and the heterozygous mutation c.2492G > A (p. Arg831Gln) in exon 10 of the *COG1* gene that was inherited from the father. The genes interacting with *COG1* were mainly involved in the transport and composition of the Golgi. The clinical data and laboratory results from a patient diagnosed with CDG-IIg were analyzed, and the causative gene mutation was identified by high-throughput sequencing. The genes and signal pathways related to *COG1* were analyzed by Gene Ontology and Kyoto Encyclopedia of Genes and Genomes enrichment analyses.

**Conclusions:**

The c.2492G > A (p. Arg831Gln) mutation in exon 10 of the *COG1* gene may be a potential pathogenetic variant for CDG-IIg. Because of the various manifestations of CDG in clinical practice, multidisciplinary collaboration is important for the diagnosis and treatment of this disease.

**Supplementary Information:**

The online version contains supplementary material available at 10.1186/s12887-021-02922-7.

## Background

Congenital disorders of glycosylation (CDG) are a group of metabolic diseases with clinical and genetic heterogeneity [1]. All the CDG types are autosomal recessive, and they are characterized by defects in glycoprotein synthesis, particularly defects in N- and O- terminal glycosylation [2, 3]. Since the first report of CDG by Jaeken et al. in 1980 [1], more than 150 types of CDG have been reported so far. CDG often involves multiple systems, including the cardiovascular system, digestive system, skeletal system, hematopoietic system and central nervous system [2, 4], and has various clinical manifestations. It has been reported that CDG-Ia caused by *PMM2* gene deficiency is the most common type of CDG [5].

Conserved oligomeric Golgi (COG) complex is a hetero-octamer complex composed of eight subunits (COG1–8). Its function is to regulate transport in the Golgi in eukaryotic cells and the integrity of the Golgi [6]. The eight subunits of COG are divided into two lobes, A (COG2–4) and B (COG5–7), and two subunits, COG1 and COG8, that bridge the two lobes [6–9]. CDG-IIg is a rare type of CDG that is caused by *COG1* deficiency, and its clinical features are multiple system damage, growth retardation, hypotonia, psychomotor retardation, cerebellar atrophy, and recurrent infections. At present, there are only three reported cases of CDG-IIg [6, 10].

In the current report, the clinical features of a rare case of CDG-IIg caused by *COG1* gene mutation are described. Additionally, genetic analysis was performed to identify the associated genes and shed light on the potential underlying mechanism.

## Case presentation

A 6-month-old Chinese male child was admitted to our department with recurring episodes of hypoglycemia for 6 months. His parents were healthy and not consanguineous, and his family history was not notable. The child was first admitted to the hospital because of repeated cyanosis and poor reaction at the age of 2 days. He was diagnosed with hypoglycemia based on his blood glucose level (which was 0.3 mmol/L) and recovered after receiving glucose and glucocorticoid infusion.

At the age of 22 days, the infant appeared pale and had a poor reaction and reduced muscle strength after fasting for 4 h for Magnetic resonance imaging (MRI) examination. His blood glucose was 2.06 mmol/L and he was recovered from glucose infusion again. Subsequently, the child showed increased muscle tension and movement retardation.

At the age of 4 months, the child experienced repeated vomiting and diarrhea for one day, and hypoglycemia reappeared (blood glucose, 4 mmol/L). In addition, the child had an episode of convulsions that lasted for 2–3 min. After intravenous infusion of glucose, blood glucose returned to normal.

At the age of 6 months, the child was admitted to the hospital for a systematic examination. Physical examination at admission showed that his body weight was 6600 g (3th centile); body length, 76.0 cm (97th centile); head circumference, 41.0 cm (3th centile); chest circumference, 43.0 cm (50th centile). He was conscious and had a good mental reaction, nutrition levels, and skin elasticity. He had strabismus in both eyes, but it was prominent in the left eye. He was unable to use the support of his elbows to get up from a reclining position, and when he stood upright, his head was tilted to one side. He could not turn over or bear his weight. He leaned forward fully in the sitting position and flexed his legs in the standing position. He was not actively conscious of grasping objects. He could keep holding objects, but he could not change objects with his hands. He could pronounce vowels, but he was insensitive to locate the sources of sound or light. He had normal muscular strength in both upper limbs, but greater muscular strength in both lower limbs, mainly in the adductor muscles. The testes were not completely lowered into the scrotum, and the result of the transillumination test was negative.

The results of laboratory tests, including blood routine (platelet count, 435 × 10 9/L; hemoglobin, 107 g/L), thyroid function, biochemical tests, stool and urine routine, and blood lactic acid, were normal. In order to rule out other possible causes of hypoglycemia, we had conducted a fasting test. The test, sustained for 4 h, was considered completed when the blood glucose level of patient indicated the hypoglycemia. The results showed that both insulin and cortisol were within the normal range, which could exclude cortisol deficiency and hyperinsulinemia. (Table 1) Computed tomography (CT) of the brain indicated that the brain parenchyma was scattered with low-density shadows, and the bilateral ventricles appeared plump.

**Table 1:** The biochemical measurements of fasting study

Characteristics	Results	Range
FPG (mmol/l)	2.2	2.2–7.0
COR (nmol/l)	425	138–698
INS (mIU/l)	0.7	<28.4

*FPG* fasting plasm glucose, *COR* cortisol, *INS* insulin

MRI of the brain indicated extensive abnormal signals in both cerebral hemispheres. This was considered to be indicative of atrophic cystic changes caused by encephalomalacia. Electroencephalogram (EEG) showed that multiple spikes/sharp-slow waves were synchronously or asynchronously discharged in the left and right occipital regions during both waking and sleep. A 2–3 Hz increase in δ activity was found in the occipital region, and the dominant rhythm of the occipital region was not significant in the waking state. Echocardiography was indicative of patent ductus arteriosus. The Gesell Developmental Schedules showed that the gross motor skills of the patient at 4 weeks and 3 months were equivalent to those of neonates at 0 months, and the fine motor skills were equivalent to those of neonates at 0–1 month. The Gesell Intelligence Scale showed that the ability to respond to people and the environment was equivalent to that of infants at 6 weeks (Developmental Quotient [DQ] = 35 points), while the ability to respond to physical objects was equivalent to that of infants at 3 weeks (DQ = 18 points). In addition, the patient’s gross motor skills were equivalent to that of infants at 0 weeks (DQ = 0 points); his fine motor skills were equivalent to that of infants at 2 weeks (DQ = 12 points), and the ability to communicate was equivalent to that of infants at 6 weeks (DQ = 35 points).

The study was carried out in accordance with the Declaration of Helsinki of the World.

Medical Association and was approved by the Committee of Medical Ethics of The Second Affiliated Hospital of Chongqing Medical University. Informed consent was obtained from parents. A high-throughput sequencing and genetic analysis, and Gene ontology (GO) and Kyoto Encyclopedia of Genes and Genomes (KEGG) enrichment analyses had been used.

After obtaining the parents’ consent and signatures on the informed consent form, blood samples were taken for a whole exon sequencing analysis. The results revealed the heterozygous intron mutation c.1070 + 3A > G (splicing) in the coding region of the *COG1* gene that was inherited from the mother, and the heterozygous mutation c.2492 g > A (p. Arg831Gln) in exon 10 of the *COG1* gene that was inherited from the father.

**Fig. 1:**
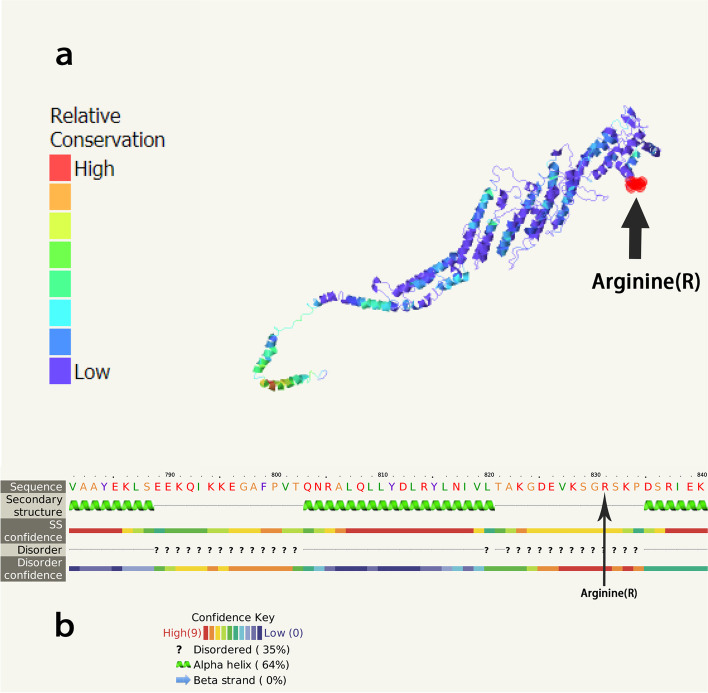
Amino acid sequence and tertiary structure of the protein encoded by the *COG1* gene. **a** Prediction of the tertiary structure of *COG* gene coding protein. **b** Secondary structure of *COG* gene encoding protein. SS, the reliability of the secondary structure. The arginine indicated by the arrow is the mutation position

The coding region of c.1070 + 3A > G in the *COG1* gene could not be predicted by the Sorting Intolerant From Tolerant (SIFT), Polymorphism Phenotyping v2 (PolyPhen_2), Rare Exome Variant Ensemble Learner (REVEL), and Mutation Taster software [6, 10]. For the coding region of the *COG1* gene, c.2492G > A (p.Arg831Gln), the results from the protein function prediction software SIFT, PolyPhen_2, REVEL revealed that it was destructive, unknown, or benign, and has not been reported in Human Gene Mutation Database (HGMD). The prediction software Mutation Taster indicated that it was a pathogenic mutation [11]. The results of other prediction software are shown in Table 2 [12–17]. The secondary and tertiary structures of *COG1*-encoded proteins were predicted by the Phyre2 software (Fig. 1) [18]. Based on the identified heterozygous mutation in exon 10 of the *COG1* gene and the patient’s clinical manifestations, we speculated that CDG-IIg in the patient may be caused by the compound heterozygous mutation.

**Table 2:** The predictions for *COG1* mutations using various software

Software	c.1070 + 3A > G (splicing)	c.2492G > A (p.Arg831Gln)
VarSome	Likely Benign	Uncertain significance
DANN	Score:0.8675	Score:0.9912
dbscSNV	ADA Score:0.001334	N/A
Mutation Taster	N/A	Disease-causing
Mutation assessor	N/A	Medium
FATHMM-MKL	N/A	Damaging
LRT	N/A	Deleterious
DEOGEN2	N/A	Tolerated
EIGEN	N/A	Pathogenic
EIGEN PC	N/A	Pathogenic
SIFT	N/A	Damaging
SIFT4G	N/A	Damaging

c. 1070 + 3A > G, the nucleotide of 1070 + 3 in the coding region was mutated from adenine to guanine; c.2492G > A, the nucleotide of 2492 in the coding region was mutated from guanine to adenine; p.Arg831Gln, amino acid 831 changed from arginine to glutamine

**Fig. 2:**
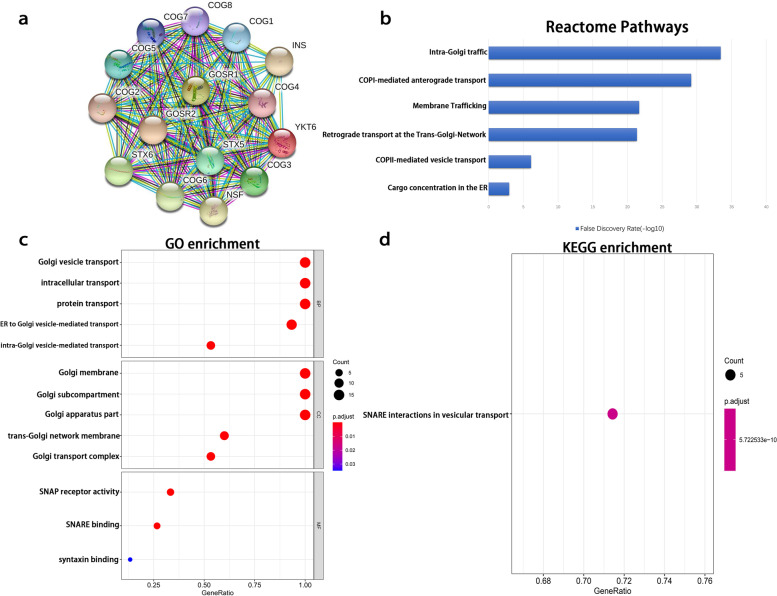
Results of KEGG, GO, and Reactome enrichment. **a** Protein-protein interaction (PPI) network. **b** GO analysis. **c** KEGG analysis. **d** Reactome enriched pathways. **b-c** The X-axis represents the ratio of involved genes, and the Y-axis represents the GO and KEGG terms. Each bubble represents a term. The size of the bubble indicates the number of involved genes. More red color indicates the smaller *p* values. The longer bar indicates the more reliable pathways. **d** The X-axis represents the false discovery rate and the Y-axis represents the pathway terms. The longer the bar the more reliable the pathways

To explore the relationship between *COG1* and related genes signal pathways, we used the Search Tool for the Retrieval of Interacting Genes database (STRING, 11.0 version) [19]. We constructed the protein and protein interaction (PPI) network of COG1 by using the search tool of the STRING database. An interaction score of 0.9 and a maximum interaction number of 50 were considered as the cut-off criteria (An additional figure file shows this in more detail (see Additional file 1)). The number of genes interact with will be more if the degree of a gene is larger. In the current study, we rank the genes from high to low. High-degree genes in the top fourteen and *INS* gene were identified as the core genes. We used core genes to perform enrichment analysis and constructed PPI network again. The PPI network is shown in Fig. 2a.

GO and KEGG enrichment analyses were performed using the Cluster profiler package [20]. The biological processes (BP), molecular functions (MF), and cellular components (CC) associated with these genes were identified, and *p* values <0.05 were considered to indicate statistical significance. In addition, we used the STRING database to perform Reactome pathway enrichment analysis [21]. A false discovery rate (FDR) of <0.05 was considered to indicate statistical significance. The results of the GO analysis are shown in Fig. 2c. With regard to BP, the COG1-related proteins were involved in Golgi vesicle transport, intracellular transport, protein transport, ER-to-Golgi-vesicle-mediated transport, and intra-Golgi vesicle-mediated transport. With regard to CC, the interacting proteins were predominantly enriched in the Golgi membrane, Golgi subcompartment, Golgi apparatus part, trans−Golgi network membrane and Golgi transport complex. With regard to MF, the interacting proteins were involved in soluble N-ethylmaleimide-sensitive fusion attachment proteins (SNAP) receptor activity, soluble NSF protein attachment protein receptor (SNARE) binding and syntaxin binding. The results of KEGG analysis showed that *COG1* was mainly enriched in SNARE interactions in vesicular transport (Fig. 2d). To further identify the signal pathways related to COG1, we used the Reactome pathway database for screening, with FDR < 0.05 as the search condition. The top six identified items are shown in Fig. 2b. The identified proteins were involved in intra-Golgi traffic, COPI-mediated anterograde transport, Membrane Trafficking, Retrograde transport at the Trans-Golgi-Network, COPII-mediated vesicle transport, and Cargo concentration in the ER.

## Discussion and Conclusions

In this report, we describe a rare case of CDG-IIg that was associated with a compound heterozygous autosomal recessive *COG1* mutation. To the best of our knowledge, there are no other reports of CDG-IIg cases in China. Three cases have been reported in other countries, and two were caused by an intron mutation in the *COG1* gene.

The patient in the present report had recurrent cyanosis, poor reaction, and hypoglycemia on the second day after birth. During hospitalization, the blood glucose was too low to be detected, and the patient was diagnosed with hypoglycemia. After discharge from the hospital, the patient had several episodes of hypoglycemia and gradually began to exhibit clinical manifestations of CDG-IIg, such as lower limb muscle strength enhancement and retardation, and epilepsy. Gene analysis confirmed that the patient had a compound heterozygous mutation in the *COG1* gene. Therefore, the diagnosis of CDG-IIg was confirmed. Further spectrum analysis showed that the CDG-IIg had an autosomal recessive inheritance pattern.

**Fig. 3:**
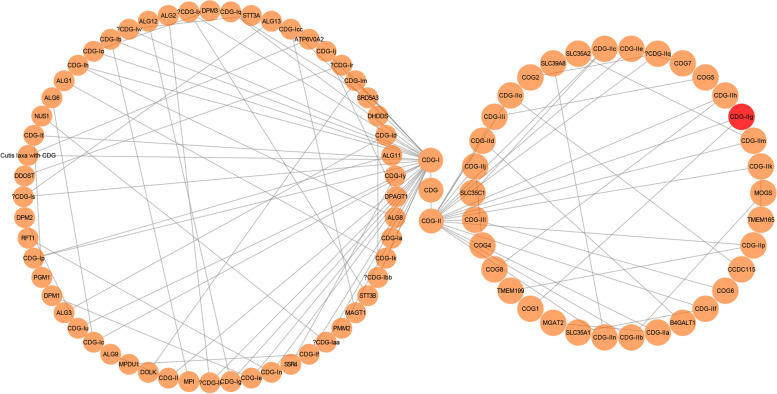
Different genotypes of CDG. The red node indicates the CDG type of patient

In eukaryotic cells, proteins have biological roles after glycosylation. Glycoproteins are composed of glucose chains and polypeptides, which can be divided into O-glycosidic bonds (that bind the serine or threonine residues of proteins) or N-glycosidic bonds (that bind the asparagine residues of proteins). At present, most types of CDG are caused by protein N-glycosylation disorder, while a few are caused by protein O-glycosylation disorder [22]. Glycosylation of N-glycosidic bonds has an important impact on the post-translational modification and function of many biological enzymes [23]. Therefore, the clinical manifestations of CDG are various. It may involve multiple organs and systems or only a single organ, and may even present with no obvious clinical manifestations. That is, the severity of CDG varies. At present, there are about 150 types of CDG, most of which exhibit neuromuscular involvement, including global developmental delay, speech delay and so on [24] CDG-I is mainly caused by a deficiency in polysaccharide synthesis or transfer in the cytoplasm and endoplasmic reticulum (ER). CDG-II is mainly caused by processing and maturation defects of the ER and Golgi polysaccharides [6]. (Fig. 3, drawn by Cytoscape [25]).

In CDG-IIg disease, Oka et al. [26] found that the steady-state level of the GEARs (a set of COG-sensitive, integral membrane Golgi proteins, including mannosidase II, GOS-28, GS15, GPP130, CASP, giantin, and golgin-84) subset is dependent on the root complex. The loss of any subunit in the COG complex may change the level of GEARs and their distribution in the Golgi complex, and this results in the biological synthesis of polysaccharides [7]. The *COG1* gene, located on chromosome 17 (17q25.1), contains 14 exons and encodes 980 amino acids. In patients with *COG1* deficiency, the levels of α-mannosidase II and β-1,4-galactosyltransferase I are significantly decreased in the Golgi matrix [6]. Any defect of the COG1 subunit will affect the function of the COG complex, thus destroying the balance of GEAR transportation and dislocation in the glycosylation process [27]. This pathological change maybe the root pathogeny for COG1. However, the connection between this pathological change and symptoms is still unclear.

In the present report, the results of bioinformatics analysis indicated that the proteins interacting with COG1 were mainly enriched in various transport pathways of the Golgi; this is consistent with the findings of previous reports. We suspect the impact of *COG1* mutation was displayed on the disorder of Golgi transport function. Meanwhile, we also speculate that *COG1* gene mutations may directly or indirectly affect cell biological processes through other genes, and this may result in the occurrence of hereditary disease. In the PPI network, we observed a relationship between COG1 and insulin. Therefore, we speculated that the observed hypoglycemia may be related to the promotion of insulin secretion by the *COG1* mutation. A search of the literature showed that there were early reports of hypoglycemia in patients with congenital glycation disorders. It was described as hyperinsulinemic hypoglycemia and was the main symptom of CDG 1b patients [28]. In addition, hypoglycemia has also been reported in patients with CDG 1a and CDG 1d [29, 30]. Because of two obvious symptoms including hyperinsulinemic and hypoglycemia, reasonable conjecture, defective glycosylation of sulphonylurea receptor, was put forward due to the confirmation of sulphonylurea receptor to be a glycoprotein [29]. However, the exact mechanism of hypoglycemia in CDG patients is still unclear. In addition, patients with PGM1-CDG (PGM1, phosphoglucomutase-1) have also been reported to have hypoglycemia [31]. PGM1 is a key enzyme in glycolysis and glycogen production. Hypoglycemia in patients with PGM1 deficiency may be related to insufficient glycogen release or abnormal glycosylation of proteins [32]. However, the patient in the present case was diagnosed with CDG-IIg and had normal serum insulin levels. Therefore, there may be other mechanisms involved in the occurrence of hypoglycemia in this case except for the connection with *INS*. Further studies involving basic research are still needed to verify our conjecture.

In two of the previously reported cases of CDG-IIg, the first patient’s brain MRI showed mega-cisterna Magna and a hypoplastic vermis. The clinical manifestations included microcephaly, bilateral macular lesions, a short neck, and widely spaced nipples. The second patient showed atrophy of the temporal cortex and floculonodular lobe of the cerebellum, together with dilatation of the lateral ventricle and fourth ventricle and cisterna magna. The clinical manifestations were shortening of the long bones, dysmorphic facial features, ulnar deviation of the fingers, thoracic scoliosis, hypospadias-I, and left-side cryptorchidism. In another reported case, the patient showed mild left ventricular hypertrophy with abnormality of diastolic function of the left ventricle. The clinical manifestations were generalized hypotonia and growth retardation. In the present case, the patient mainly showed growth retardation, convulsion, and hypoglycemia, which is a new symptom of CDG-IIg. Because CDG is extremely rare, its diagnosis depends on clinicians’ awareness and vigilance. When the above clinical manifestations, especially those involving the nervous system, cannot be explained by other diseases, the possibility of CDG should be considered [24].

At present, serum transferrin isoelectric focusing (IEF) is still a common method for the diagnosis of CDG, but the IEF results of some CDG patients can be normal. Gene technology (whole exon and genome sequencing), enzyme analysis, and glycomics are emerging as reliable methods in the diagnosis of CDG [33, 34]. In the present case, CDG was diagnosed based on high-throughput sequencing and genetic analysis. With regard to treatment, no specific treatment for CDG-IIg has been reported so far, and symptomatic treatment is widely used in the clinic. In the present patient, intravenous infusion of glucose solution was used to correct hypoglycemia. What’s more, body rehabilitation was also applied for the improvement of growth retardation due to the hereditary nature of this disease. Similarly, the treatment of specific symptoms has been reported for other types of CDG. PGM1-CDG patients can be treated with oral galactose or nutritional therapy, which is known to be effective [35]. Additionally, stem cell transplantation can correct neutropenia and lymphopenia in patients with PGM3-CDG (PGM3, phosphoglucomutase 3) [36], and patients with phosphomannose isomerase deficiency (MPI-CDG) can be treated by mannose and liver transplantation [37]. However, CDG is a chronic genetic disease that often involves multiple organs and systems. Therefore, patients with CDG need to be managed and treated systematically. In addition, recombinant enzymes, drug chaperones, active sugar compounds, and gene therapy might have potential as effective therapeutic methods in the future [1, 38]. Importantly, genetic screening is performed in the families of patients to ensure early and rapid diagnosis and, thus, improve prognosis. Furthermore, multidisciplinary collaboration is important for the diagnosis and treatment of this disease.

## Supplementary Information

**Additional file 1: Supplementary Figure 1.** Description of data: Protein-protein interaction (PPI) network, which can be obtained from STRING database through keyword *COG1*. An interaction score of 0.9 and max number of interactors of 50 were considered a cut-off criterion. The *INS* gene is pointed by a black arrow.

## Data Availability

The datasets used and/or analyzed during the current study are available from the corresponding author on reasonable request.

## Abbreviations

CDG: Congenital disorder of glycosylation
COG1 (gene): Component of oligomeric golgi complex 1
COG (protein): Conserved oligomeric golgi
SNARE: Soluble NSF attachment protein receptor
NSF: N-ethylmaleimide-sensitive fusion protein
PMM2: Phosphomannomutase-2
MRI: Magnetic resonance imaging
CT: Computed tomography
EEG: Electroencephalogram
DQ: Developmental quotient
SIFT: sorting intolerant from tolerant
PolyPhen_2: Polymorphism phenotyping v2
REVEL: Rare exome variant ensemble learner
HGMD: Human gene mutation database
STRING: Search tool for the retrieval of interacting genes database
PPI: Protein and protein interaction
GO: Gene ontology
KEGG: Kyoto encyclopedia of genes and genomes
BP: Biological processes
MF: Molecular functions
CC: Cellular components
FDR: False discovery rate
ER: Endoplasmic reticulum
SNAP: Soluble NSF attachment proteins
GEARs: A set of COG-sensitive, integral membrane Golgi proteins
PGM1: Phosphoglucomutase-1
IEF: Isoelectric focusing
