# Supplementary material for: Component of oligomeric Golgi complex 1 deficiency leads to hypoglycemia: a case report and literature review
Source: BMC Pediatr. 2021 Oct 8;21:442. doi: 10.1186/s12887-021-02922-7 (PMC8499485; doi:10.1186/s12887-021-02922-7)
Supplement: Supplementary file 1 — Additional file 1: Supplementary Figure 1. Description of data: Protein-protein interaction (PPI) network, which can be obtained from STRING database through keyword COG1. An interaction score of 0.9 and max number of interactors of 50 were considered a cut-off criterion. The INS gene is pointed by a black arrow. [file 12887_2021_2922_MOESM1_ESM.docx]

**Figure S1**


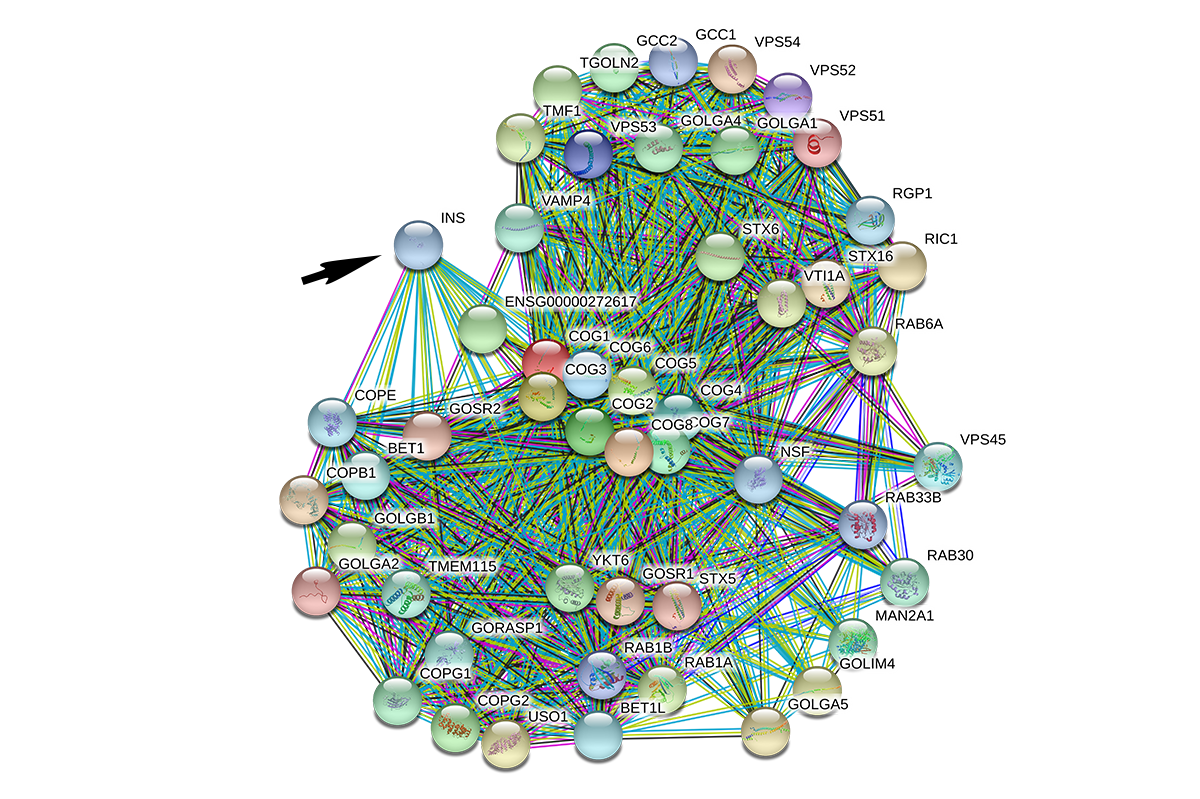


**Supplementary Figure 1** Protein-protein interaction (PPI) network, which can be obtained from STRING database through keyword COG1. An interaction score of 0.9 and max number of interactors of 50 were considered a cut-off criterion. The INS gene is pointed by a black arrow.
